# Mr. Ward, on Hydrophobia

**Published:** 1807-04

**Authors:** Michael Ward

**Affiliations:** Manchester


### *To the Editors of the Medical and Phyſical Journal.*

Gentlemen,

At any time, but particularly at a criſis like the preſent, when ſo many perſons in different parts of the kingdom have been recently bitten by mad dogs, (though I believe the reports have been exaggerated); the ſucceſsful iſſue of a caſe of *genuine* hydrophobia could not fail to prove in the higheſt degree intereſting, as well as gratifying, nor would any one rejoice more ſincerely on ſuch an occaſion than myſelf. I cannot help thinking, however, that this great deſideratum will be moſt ſpeedily and effectually promoted, by exerciſing ſuch a degree of diſcernment as will enable us to diſtinguiſh what is *genuine* from what what is *ſpurious*; in which light I apprehend we muſt view the caſe publiſhed by Mr. Hicks in your laſt number.

I admit that the patient, William Miles, had been bitten by a *ſuppoſed* rabid dog;* he alſo appears to have had an averſion, real or *imaginary*, to water;† as well as a tendency to bite, an occurrence which certainly happens ſometimes in true hydrophobia: but in every other reſpect, the ſymptoms were the reverſe of thoſe which generally take place in that diſeaſe, and they alſo appear to have been very much aggravated by the influence of *terror* operating upon a weak imagination. For inſtance, when his mother aſked him to drink ſome beer, he replied, "Drink! no! I have been bit by a mad dog, *and immediately left the houſe.*"

A reply, which ſeems rather to have been dictated by what he imagined was *uſual in ſuch caſes*, than by that ſtrong and peculiar *dread of liquids*, the bare mention of which is in general ſufficient, (perhaps in 99 caſes out of 100) to produce the moſt violent ſpaſms; inſtead of which we are told, 'he left the houſe;' and ſoon after committed ſuch extraordinary acts of violence, as none but a *maniac* would have attempted.

"At a ſmall diſtance from the houſe, he met two men, *both of whom he ſhook and bit.* Soon after he paſſed out of the road, *through a thick hedge*, into a cloſe, which he croſſed, and being ſtopped by ſome *ſtrong oak railing*, he graſped it, and violently ſhaking it, broke it. He then went into a farm yard, *and laid himſelf down in the corner of a cart hovel*; he could not lie long, but ſoon began to roll about. His mother having followed him, looked into the cart hovel; he begged her to keep from him, as from his diſtreſſed feelings, he could not anſwer for what he did, upon which ſhe ran away. He ſoon came out, and croſſing the farm yard, *attacked a bullock*, after which he went towards the houſe, which is at ſome diſtance from the yard.

"In the porch of the houſe was lying a large ſavage maſtiff dog; the dog came out of the porch at him, but before it approached half way towards him *it turned back and ran into the porch; he followed the dog, attacked it, got it down, and from his attitude, a young man who was looking on ſuppoſes he attempted to bite it*; *the dog got from under him, and ran away.*"

Whoever

---

* Whether the dog was really mad, does not ſufficiently appear, as we are not informed whether any other perſons were bitten by him, or what became of him.

† It is extraordinary that he had no averſion to the medicine, which we are told was taken regularly.

Whoever has paid the leaſt attention to the ſubject of Hydrophobia Rabioſa, will immediately perceive a ſtriking difference between the ſymptoms here deſcribed, and thoſe of the diſeaſe in queſtion; and which afford poſitive proof, in my mind, (when taken in conjunction with other circumſtances to be conſidered by and by) that the diſeaſe was neither more nor leſs than a ſlight degree or ſpecies of tetanus, accompanied by an unuſual affection of the brain, eaſily traced to terror acting upon an irritable, *and probably melancholic*, temperament; and what tends to confirm this opinion is, that though he is ſaid to have had an averſion to the ſight of water, yet it does not appear that he had any averſion to the ſight of other liquids. Mr. Hicks alſo tells us that he took the medicine, conſiſting of camphoric mixture, &c. *regularly*, *till he found himſelf better*.

The other circumſtances to which I have alluded, are,

1. The ſhort ſpace of time which intervened, (namely, *ten days*) between the bite and the acceſſion of the diſeaſe;* a circumſtance ſo unprecedented in the Hiſtory of Hydrophobia, as to afford the moſt ſatisfactory evidence againſt the *rabid*, and in favour of the *tetanic*, origin of the diſorder.

2. Another circumſtance which happened in the preſent caſe, and which is equally unuſual in hydrophobia, is, that "*of the throat of the patient projecting out almoſt to his chin.*"

Nor, 3, has my reading (which has been pretty extenſive on this ſubject) or experience, furniſhed me with a ſingle inſtance of hydrophobia in which an equal degree, or any thing like it, of nervous ſympathy ſubſiſted between the bitten part upon preſſure being applied, and the ſuperior parts of the body, as took place in the caſe of Miles; or in which the patient experienced the ſenſation of being *bound with ropes*, (though it is very common in tetanus) as expreſſed in the following paſſages. "Upon touching it with his finger, *he immediately complained of the tightneſs of his ſtomach and throat being increaſed*. This induced me to touch the part two or three times with my finger, *and the ſpaſmodic affection of the præcordia and fauces became more or leſs increaſed, according to the degree of preſſure on the part*." "The ſpaſmodic affection of the præcordia and fauces being evidently increaſed by *the ſlightest preſſure* on the inflamed cicatrix, I was induced to apply a cauſtic to it." "On examining his leg I found the cauſtic that was applied on the Wedneſday, had had the deſired effect, and produced a large and good

* He was bitten on the 19th of November, and began to complain on the 29th.

good efchar; *upon touching which* he complained of the fpafmodic affection of the præcordia, &c. being increafed. To ufe his own words, *by touching the part, the cords or ropes with which he felt as if he was bound were tightned.*"

4. It appears by the patient's own account, that he was expofed to fuch a degree of *cold and moisture* on Sunday, November 30, as was fufficient, *without fuppofing the dog to have been mad*, to account for all, (and much more than all) that followed. For we muft recollect, that the patient was at this time in a frame of body and mind, which rendered him peculiarly fufceptible of thofe morbid trains of action which afterwards occurred, having lately received a wound or puncture on the leg, *which was in an irritable painful state*; *the pain and ftiffnefs extending to the knee and thigh*; a degree of fever had fupervened: there was alfo a confiderable degree of mental derangement, which is very unufual *in the early ftage* of hydrophobia: and it is well known by every perfon who is at all converfant with medicine, that nothing is more common than for tetanus, with all its dreadful train of fymptoms, (compared with which thofe of Mr. H's patient are fcarcely worth mentioning), to enfue from a much lefs degree of expofure to cold, than that which evidently took place in the prefent inftance.* The proofs are as follow.

" Upon afking him, (fays Mr. H.) if he could call to mind the day on which the dog bit him, and in what way the wound went on, he informed me that it was on the Wednefday week before he was taken ill, which was November 19th; that after he got home, his leg fmarting, he put down his ftocking to look at the part; it had bled a drop of blood, which coagulating, *ftuck his ftocking to it.* As the place was but fmall, *he did nothing to it*, nor faid any thing about it. It healed up, as he fuppofed well, with a fmall dry fcab on it, *which in five or fix days was rubbed off.* It continued to look well till the Thurfday week, November 27th, *when it itched and appeared rather red.* On the Friday, November 28, *he felt flight prickling pains in it, and the rednefs increafed.*

On Saturday, November the 29th, *the pain in the part increafing, about noon his knee became ftiff, and in the courfe of the afternoon the ftiffnefs, with fome pain, extended up the inner fide of his thigh. In the evening, about ten o'clock, he was taken with great coldnefs*, fo much fo, that he crept down into his bed fo as to

* See a cafe of tetanus, related by Mr. Nayler, of Gloucester, in the Medical and Phyfical Journal, vol. 10. p. 492.

to cover his head with the bed clothes; and, as he expreſſed himſelf, he could not bear the cold wind to come to his face.

On the Sunday morning, as ſoon as he was up, the ſenſation of coldneſs increaſing, he went to the fire, and in eating his breakfaſt, *found his throat ſtiff, and ſwallowed with difficulty.*

*Soon after breakfaſt he left his maſter's houſe, in what way he knew, not, feeling himſelf hurried. He recollects falling down in a field,* and on getting up, was taken with pain and ſtiffneſs at the pit of his ſtomach, which extended up to his throat, as if he had been bound with a ſtrong cord. He now became more hurried, and knew not where he went, having, from his diſtreſſed feelings, a very imperfect idea of what followed, till after he was ſecured, when his reaſon returned.*

It was alſo during this ramble, *after he had broke the ſtrong oak railing, and before he attacked the bullock*, that " *he went into a farm yard, and laid himſelf down in the corner of a cart hovel.* How long he lay in the porch, after his engagement with the maſtiff, does not appear, I ſhall therefore lay no ſtreſs upon that point, *nor upon his laying upon a bundle of ſtraw, on the ground floor, from the time he was ſecured on the Sunday, till the time of Mr. H's firſt viſit on the Monday :* the other points, inſiſted on, being fully ſufficient to ſhow that the diſeaſe with which William Miles was afflicted, had a much greater affinity to tetanus than to hydrophobia.

I agree with Mr. H. that the cauſtic had the principal ſhare in effecting the cure,† but muſt beg leave to differ with him as to the modus operandi. He attributes the effects to its " deſtroying the extremities of the nerves that were thus excited by the morbid action that was going on in the cicatrix."

I think the effects are to be aſcribed, to its rendering the nerve, or nerves, ſituated immediately above, and communicating with the cicatrix, *incapable of tranſmitting* what I have taken

* How long he lay there is uncertain, but it is probable, from the state he was in when he got up, that it was no very short time ; but this is not very material, *the effect produced* being the point principally deserving of attention.

† Though two drachms of tinct. of assaf. half a drachm of tinct. of opium, one drachm of compound spirit of ammonia, and fourteen drachms of camphorated mixture, taken regularly every two hours, for seven days, is no very *trifling* medicine : nor is it very easy to conceive, (though no doubt can be entertained of the accuracy of Mr. H's statement), that a person who could take twenty-one ounces of tincture of assafœtida, five ounces and a quarter of tincture of opium, ten ounces and a half of compound spirit of ammonia, *and a hundred and forty-seven ounces (or upwards of nine pints ale measure)* of camphorated mixture, *in a week*, besides nourishments of various kinds; (for though no mention is made of the latter, I conclude some support of that kind must have been found necessary), could have had *either a very great difficulty in swallowing, or any very violent aversion to liquids.*

taken the liberty to call, in a publication which I have been for fome time preparing, and which is nearly ready for the prefs) THE TETANIC AURA *from the wounded part*, (*where I have endeavoured to fhow it is generated*), *to the mufcles affected with fpafm*, *fituated in the fuperior parts of the body.*

And fo far is this from being a queftion of mere curiofity, that it leads to a *practical inference*, *in the cure of hydrophobia as well as tetanus*, which will prove, if I am not greatly miftaken, of confiderable utility and importance.

There are a few other points in which I have the misfortune to differ in opinion with Mr. H. the principal of which are as follow:

Speaking of hydrophobia, he fays, "the cicatrix of the wound is *always* inflamed, prior to the fyftem being affected."

Doubtlefs this does fometimes happen, but did not occur in any of the three cafes which I have feen; and many other inftances might eafily be adduced, did the cafe require, or the time permit.

Mr. Hicks then adds, "But after the cicatrix has become inflamed, and the fyftem affected, fuppofing abforption to have taken place, *the application of a cauftic is not thought of.*"

That the application of cauftic, after the cicatrix has become inflamed; and the fyftem affected, has not merely been thought of, but actually recommended, is very clear, and will be eafily proved. (I have alfo been told, from good authority, that it has often been practifed).

For this purpofe, we need only confult Zoonomia, cl. iii. 1. 1. 15. and the Med. and Ph. J. v. ii. p. 547, where, (without knowing that this practice had been previoufly advifed, or even thought of by any other perfon) I have expreffed myfelf in words to the following effect. *After the commencement of the difeafe, the wounded part being painful, and excifion not having been performed, I fee no impropriety in deftroying the life of the part, by applying a pledgit fpread thick over with the calx cum kali pura, and a poultice afterwards.*"

The hafte with which the above remarks on Mr. Hicks's communication have been drawn up, (owing to my not having feen your laft number till the 9th inft.) may poffibly have led me into miftakes of equal importance with thofe upon which I have taken the liberty to animadvert. Should this prove to be the cafe, I can only fay, that I fhall be as ready to acknowledge and correct as I have been to advance them. To the fame caufe is to be attributed the neceffity I am under of thus abruptly fubfcribing myfelf,

Yours, &c.

MICHAEL WARD.

*Manchester, Nov. 15, 1807.*

P. S. In the prefent alarming emergency, it may perhaps be excufable fo far to anticipate my intended publication, as to mention briefly *the plan of cure* which will there be recommended, and which *I have long since conceived*, as the refult of a regular train of facts and arguments, as what appears to me the beft that can be adopted in Hydrophobia and Tetanus, but particularly in the former; agreeing in many refpects, but differing materially in others, with that which I had the honour to propofe *nearly three years ago*, in an Effay on thefe difeafes, which was inferted in the 11th vol. of the Med. and Phys. Journal, p. 538—548.*

The rationale of the above mentioned plan of cure, is in part as follows.

Sect. VI. "The exuberance and retrograde action (IV.)† originate in the wounded or lacerated part, and are propagated from thence along the nerves, by means of what I fhall take the liberty to call, THE HYDROPHOBIC, OR TETANIC AURA, to the mufcles affected with fpafm."

Sect. VII. "The retrograde motion of the *aura*, (VI.) is manifefted by a fenfe of uneafinefs or pain, commencing in the injured part, which is immediately fucceeded by fhooting pains proceeding upwards, from thence to the diaphragm, the vifcera of the abdomen and thorax, mufcles of deglutition, refpiration, &c.; thefe being followed by an immediate and often violent increafe of the fpafms."

Sect. X. "It appears to be chiefly through the medium or the fympathetic and recurrent nerves, the par vagum, and the branches of thefe which communicate with the other nerves, which fupply the vifcera of the abdomen and thorax, that the hydrophobic and tetanic aura has accefs to the parts moft affected with fpafmodic and retrograde action; as the œfophagus, trachea, mufcles of deglutition, and of refpiration; the heart, ftomach, inteftines, abdominal mufcles, &c. &c. There is alfo great reafon to believe, that the recurrent nerves are the principal agents employed in producing the characteriftic fymptom of hydrophobia, a dread of water."

INDICATIONS OF CURE.

"The indications of cure, according to the above hypothefis, will be, 1. *To intercept the* AURA, *or retrograde nervous impulse*

---

* The evident connection between the principles laid down in the following extracts, and those laid down in the Outlines published in your Journal in June 1804, may be seen by comparing them with each other.

† The proximate cause, (and many other particulars) was pointed out in the *Outlines* already referred to, and which the Reader is requested to peruse in this stage of the discussion. M. & P. J. v. 11. p. 538.

*pulſe*, (VI.) *in its paſſage from the injured part, to the centre and ſource of the nervous ſyſtem.* 2. To appeaſe the tumult in that part of the nervous ſyſtem, ſituated in the anterior, poſterior, and lateral parts of the neck and throat, from the clavicles upwards, as high as the ears and chin. 3. To reſtore the equable and regular diſtribution of the ſenſorial power, amongſt the different claſſes of nerves, muſcles, &c. And, 4. To reſtore *the natural*, that is, *the* PROGRESSIVE *action*, of the muſcular fibres, generally throughout the body.

In order to fulfil the firſt, and moſt important of theſe indications, there are two methods of proceeding which I have fixed upon, as promiſing exceedingly fair to be attended with ſucceſs, in many caſes, both of hydrophobia and tetanus; the attending phyſician or ſurgeon, exerciſing his judgment as to which will be moſt expedient in each individual caſe, until experience ſhall have determined to which the preference is due.

The firſt is, to apply a cauſtic, (the calx cum kali pura, which I recommended for this purpoſe three years ago, ſeems to act moſt ſpeedily and certainly) at the diſtance of, from one to three or more inches, (according to the ſituation and courſe of the offending nerve,) *above* the bitten or wounded part, removing the eſchar, and repeating the application oftener or ſeldomer, according to the effect produced.

The other (and by far the moſt effectual where it can be ſafely practiſed) method *of intercepting the aura*, in its paſſage from the injured part to the centre, and ſource of the nervous ſyſtem, is, *to divide the nerve*, (or nerves) *which forms the medium of communication between the topical affection and the brain or ſpinal marrow.*

It may be uſeful in either caſe, in enabling us more eaſily to diſcover the nerve to be operated upon, *to apply preſſure* by means of a tourniquet, or otherwiſe, *above the part affected*; there are alſo other neceſſary precautions to be taken, many of which will readily occur to every experienced practitioner, but which, I have neither time nor room to enumerate.

To appeaſe the tumult in the nervous ſyſtem, which forms the *second* indication of cure, either the opiate frictions, *or plasters conſiſting chiefly of galbanum and opium, with a ſmall proportion of oil of amber, ſhould be applied to the ſcrobiculus cordis, neck, throat, and ſpine, and repeated twice or thrice a day.*

After having ſucceeded in this object, medicines of an oily and antiſpaſmodic nature, ſuch as a ſolution of aſſafœtida, with a ſmall proportion of tincture of opium, and oil of almonds, might be given internally with advantage.

To fulfil the third and fourth indications of cure, (ſee above) COLD WATER, applied as recommended in my former

Outlines,

Outlines, is principally to be depended upon, with the occaſional uſe (eſpecially in tetanus) of purgatives; but I entertain ſanguine hopes, that the methods of treatment pointed out above, in conſidering the proper means of fulfilling the firſt and ſecond indications of cure, will in general ſupercede the neceſſity of having recourſe to a remedy, which muſt often prove, (in hydrophobia) difficult in the application, and irkſome in the extreme.

I am fully aware of the great diſadvantages I labour under, in thus communicating, *without connection, or reſerve*, the ideas which have occurred to me, on a ſubject of ſo much difficulty and importance; to obviate which, I have only to urge the ſincerity of my motive, that of a wiſh to increaſe, to the utmoſt of my power, the reſources of medicine and ſurgery; and to requeſt your readers will ſuſpend their opinions on the ſcheme of practice I have ventured to propoſe, until the *whole* of the evidence upon which it is founded ſhall have been made public.

---

